# Cutaneous Adverse Effects of Hydroxychloroquine and Leflunomide in Connective Tissue Diseases: A Report of Three Cases and a Literature Review

**DOI:** 10.7759/cureus.85495

**Published:** 2025-06-07

**Authors:** Carina-Andreea Bazon, Elena Porumb-Andrese, Antonia Hutanu, Olguta Orzan, Daciana Elena Branisteanu

**Affiliations:** 1 Dermatovenerology, Căi Ferate (CF) Clinical Hospital, Iasi, ROU; 2 Dermatology, Grigore T. Popa University of Medicine and Pharmacy, Iasi, ROU; 3 Dermatology, Universitary Railways Hospital, Iasi, ROU; 4 Dermatology, Elias Emergency University Hospital, Bucharest, ROU; 5 Dermatology, Carol Davila University of Medicine and Pharmacy, Bucharest, ROU

**Keywords:** connective tissue disease, cutaneous adverse effect, hydroxycloroquine, leflunomide, pathophysiology

## Abstract

Connective tissue diseases (CTDs) are a group of systemic autoimmune disorders that require long-term treatment with immunomodulatory and immunosuppressive agents to control disease progression and inflammation. Among the most frequently used agents are hydroxychloroquine and leflunomide, which have proven efficacy in conditions such as systemic lupus erythematosus, rheumatoid arthritis, and Sjögren’s syndrome. The use of these drugs has been correlated with dermatologic adverse reactions of varying severity, spanning from common and mild skin eruptions to rare but severe cutaneous syndromes. This article presents three clinical cases of patients undergoing treatment with hydroxychloroquine or leflunomide who developed dermatological manifestations, such as annular erythema, mucocutaneous hyperpigmentation, and phototoxic reactions. The pathophysiological mechanisms underlying these adverse effects are discussed, and a literature review is provided to contextualize the frequency, risk factors, and management strategies for these adverse reactions. The findings emphasize the importance of early recognition and monitoring of cutaneous adverse effects in patients receiving long-term immunomodulatory therapy. Further research is needed to better understand the molecular mechanisms involved in drug-induced dermatologic side effects and to develop strategies for minimizing these risks while ensuring optimal therapeutic outcomes.

## Introduction

Connective tissue diseases (CTDs) are a group of systemic autoimmune disorders that require the use of corticosteroids, immunomodulatory and immunosuppressive agents, as well as biological therapies for treatment [1]. Cutaneous adverse effects are frequently observed during the treatment of CTDs with immunomodulatory and immunosuppressive agents such as hydroxychloroquine and leflunomide. These manifestations are diverse and include maculopapular drug eruptions, photosensitivity reactions, alopecia, skin ulcerations, pigmentary changes, urticaria, and drug-induced lupus-like syndromes [2-7]. The underlying mechanisms are complex, involving hypersensitivity reactions, direct cytotoxic effects on keratinocytes and melanocytes, and immune system dysregulation [8-12]. The clinical presentation of these adverse effects can vary from mild, transient rashes to severe, potentially life-threatening skin conditions requiring treatment discontinuation.

In the following sections, we will detail the most common cutaneous adverse effects associated with hydroxychloroquine and leflunomide use, based on both our clinical dermatology practice and current literature data.

## Case presentation

Case 1

**Figure 1:**
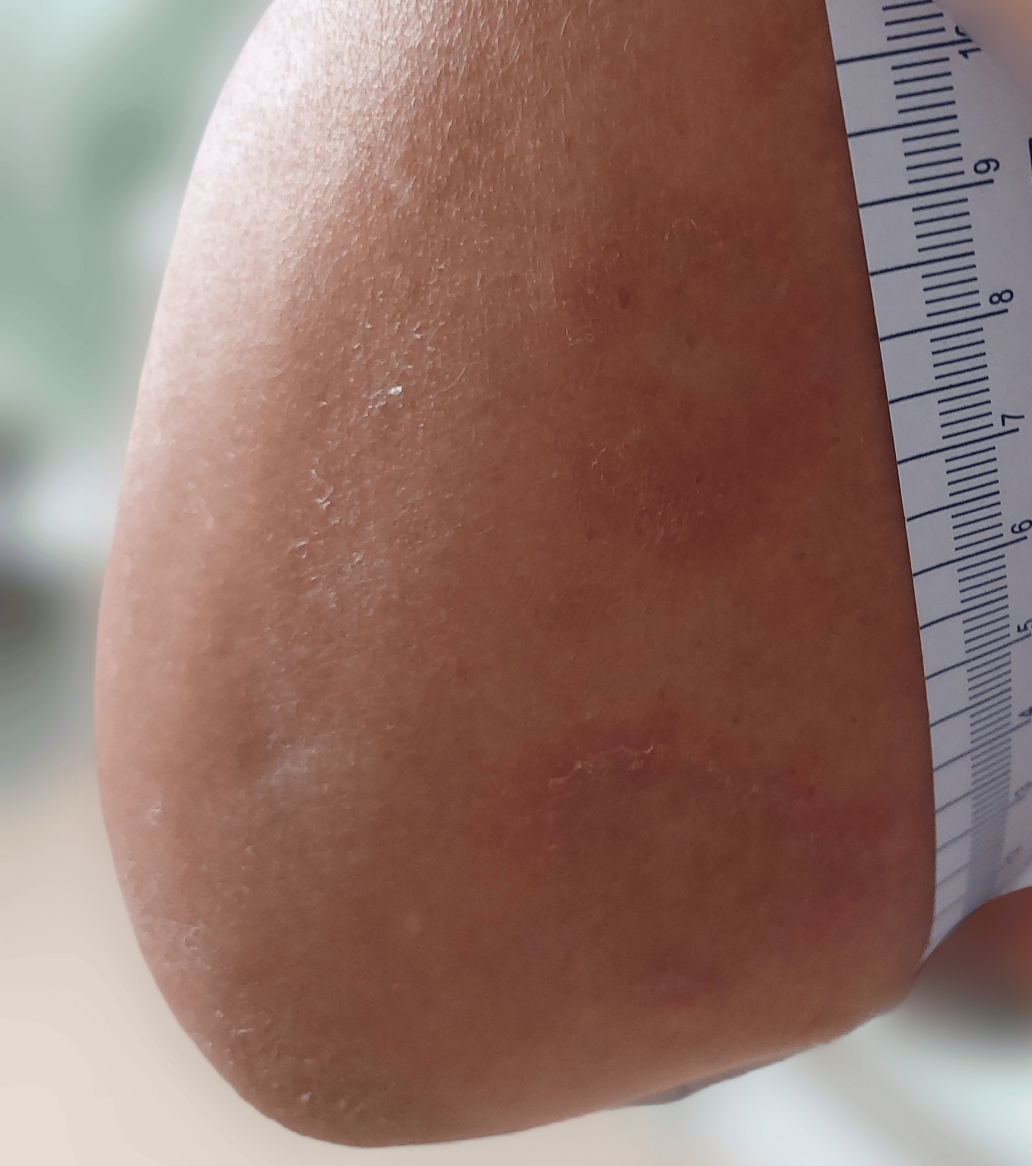
Erythematous round-oval lesions with elevated borders and a clear center, located on the forearms of a 68-year-old female patient treated with hydroxychloroquine for the past four months.

We present the case of a 68-year-old female patient diagnosed with systemic scleroderma in its diffuse cutaneous form and secondary Sjögren's syndrome. She had been receiving 500 mg of mycophenolate mofetil in progressively increasing doses since 2021 and 200 mg of hydroxychloroquine twice daily for approximately four months. She presented to our clinic with asymptomatic, erythematous, round-oval lesions with elevated borders and a clear center, located on the forearms (Figure 1). The patient reported that she had stopped taking hydroxychloroquine two weeks before her visit due to the appearance of these lesions.

Laboratory investigations revealed leukopenia with lymphopenia, mild normochromic normocytic anemia, thrombocytopenia, elevated erythrocyte sedimentation rate (ESR) and C-reactive protein (CRP), total and direct hyperbilirubinemia, hypocalcemia, elevated gamma-glutamyl transferase (GGT) levels, hypergammaglobulinemia (IgG), and asymptomatic leukocyturia with identification of *Enterococcus faecalis* sensitive to amikacin. Viral serologies for hepatitis B and C were negative. Dermoscopic evaluation was nonspecific, and histopathological examination could not be performed due to the patient's refusal of a skin biopsy.

Case 2

**Figure 2:**
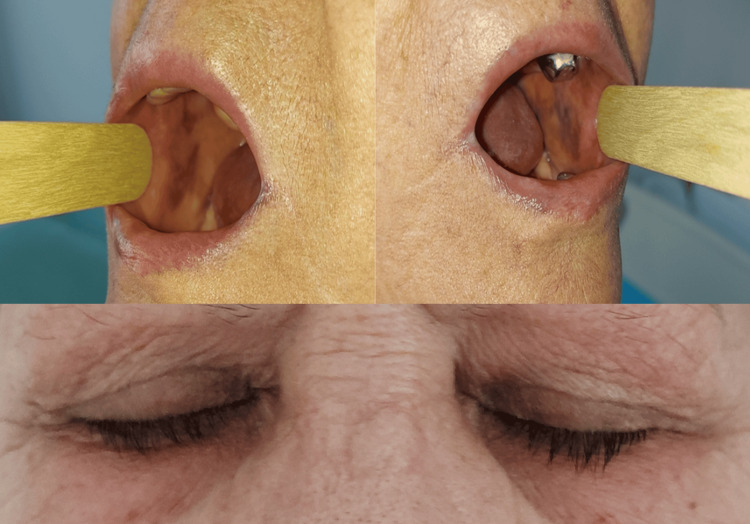
Periorbital hyperpigmentation and two irregular, well-defined, hyperpigmented, asymptomatic plaques located bilaterally on the jugal mucosa corresponding to the last molars of a 65-year-old female patient treated with hydroxychloroquine for approximately three years.

A 65-year-old female patient diagnosed with Sjögren's syndrome since 2021 and systemic lupus erythematosus since 2024 had been on 200 mg of hydroxychloroquine twice daily for approximately three years. She presented to our clinic due to persistent erosions in the oral cavity, but what caught our attention was a periorbital hyperpigmentation and two irregular, well-defined, hyperpigmented, asymptomatic plaques located bilaterally on the jugal mucosa corresponding to the last molars (Figure 2).

Laboratory findings were consistent with Sjögren’s syndrome, showing a mildly decreased C3 complement level, a slight elevation of CRP, and a mild increase in rheumatoid factor, with other routine laboratory parameters remaining within normal limits. Dermoscopic examination could not be conducted, and the patient refused to undergo a mucosal biopsy.

Case 3

**Figure 3:**
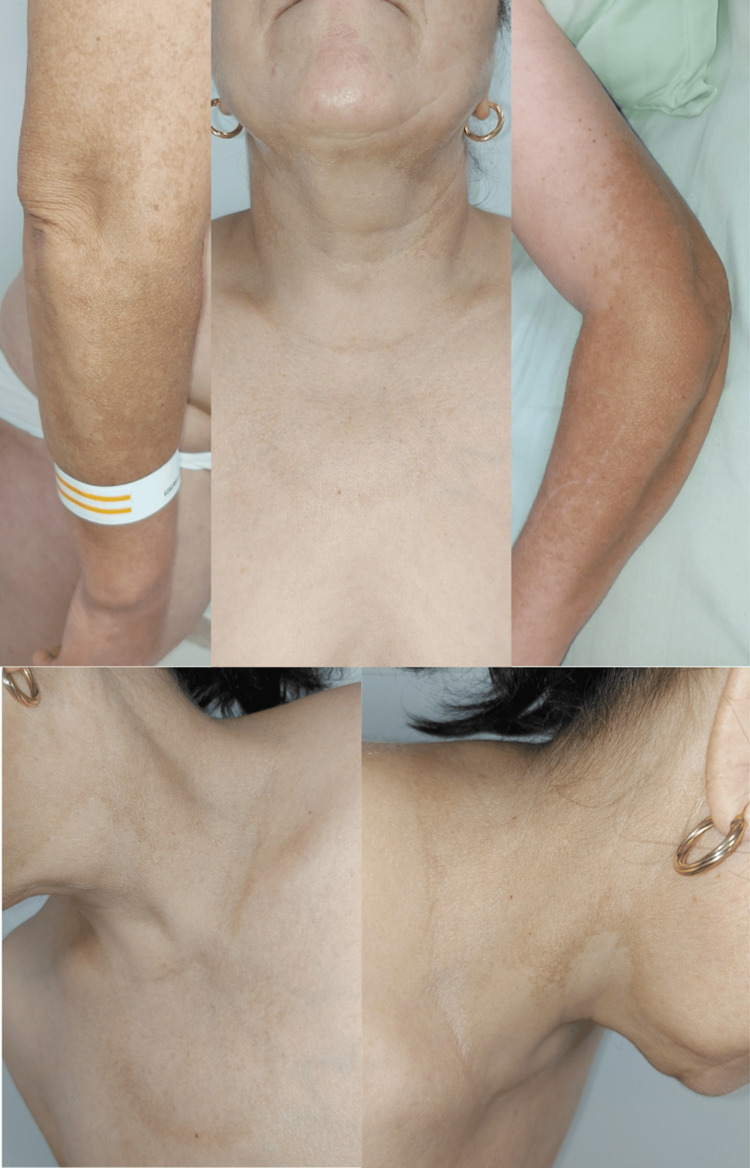
Two well-defined, irregularly bordered, hyperpigmented, asymptomatic plaques on the extensor surfaces of the upper limbs, as well as a similar plaque on the anterior cervical area extending to the lateral sides of the neck and anterior thorax of a 52-year-old female patient treated with leflunomide for four years.

A 52-year-old female patient diagnosed with rheumatoid arthritis since 2009 was undergoing treatment with Benepali 50 mg/week subcutaneous injection and 20 mg/day of leflunomide for the last 51 months. She presented to our clinic with persistent subcutaneous nodules on her lower limbs that had appeared three weeks prior. However, additional cutaneous manifestations, suggestive of a phototoxic reaction, were noted during the examination: two well-defined, irregularly bordered, hyperpigmented, asymptomatic plaques on the extensor surfaces of the upper limbs, as well as a similar plaque on the anterior cervical area extending to the lateral sides of the neck and anterior thorax (Figure 3).

Laboratory assessment identified an ongoing inflammatory syndrome (elevated ESR and CRP), mild hypocalcemia, slight azotemia, and an elevated rheumatoid factor, while all other laboratory values, including complete blood count, liver function tests, lipid profile, urinary biochemistry, thyroid function tests, immunoglobulin panel, and serum complement levels (C3 and C4), were within normal limits. Histopathological examination could not be performed due to the patient's refusal of a skin biopsy, and dermoscopic evaluation was nonspecific.

## Discussion

Leflunomide and hydroxychloroquine are among the most commonly used drugs in the management of connective tissue diseases. Leflunomide, an immunomodulator that inhibits pyrimidine synthesis, and hydroxychloroquine, an antimalarial with immunomodulatory effects, are effective in reducing inflammation and disease progression. However, their long-term use can lead to various cutaneous adverse effects [1].

Hydroxychloroquine is widely prescribed for rheumatological conditions like systemic lupus erythematosus and rheumatoid arthritis due to its immunomodulatory effects [13]. However, it has been linked to various cutaneous adverse effects, particularly in women over 50 years old [14]. The most common include mucocutaneous hyperpigmentation (10% to 30% of cases with chronic use), maculopapular or urticarial rash (10% within four weeks), and pruritus unresponsive to antihistamines (10%) [2]. Rare reactions (<1/100) include acute generalized exanthematous pustulosis (AGEP), Stevens-Johnson syndrome (SJS), toxic epidermal necrolysis (TEN), drug reaction with eosinophilia and systemic symptoms (DRESS), photosensitivity, and psoriasis-like eruptions, despite hydroxychloroquine’s photoprotective role in photodermatoses. In most cases, discontinuation leads to spontaneous resolution within weeks to months, with or without topical corticosteroids [3].

A systematic review of 94 studies identified 689 hydroxychloroquine-related dermatologic adverse effects, the most frequent being drug eruption or rash (52%), followed by cutaneous hyperpigmentation (17%), pruritus (9%), and more severe but rare reactions such as AGEP, SJS/TEN, and stomatitis [3]. Similarly, the product leaflet categorizes these effects by frequency: common (skin rashes, alopecia, pruritus), uncommon (pigmentary changes, hair bleaching), and very rare (AGEP, exfoliative dermatitis, erythema multiforme, SJS, DRESS, TEN, and photosensitivity) [4].

The cutaneous side effects of hydroxychloroquine are thought to arise from its complex mechanisms of action, which involve immunomodulation, anti-inflammatory effects, and interactions with cellular processes. While the exact mechanisms behind its dermatologic adverse effects are not fully understood, several pathways have been proposed: 1. Accumulation in melanin-rich tissues, leading to hyperpigmentation [8]; 2. Modulation of toll-like receptors and cytokines, which may trigger rashes and hypersensitivity reactions [8]; 3. Interference with lysosomal function, possibly causing lichenoid eruptions or AGEP [15]; 4. Increased oxidative stress and UV sensitivity, contributing to photosensitivity and hyperpigmentation [9]; 5. Type IV hypersensitivity reactions leading to severe conditions like SJS/TEN [10]; 6. Genetic predisposition factors that may influence the likelihood of adverse effects [16].

On the other hand, leflunomide is a disease-modifying antirheumatic drug (DMARD) used primarily for rheumatoid arthritis [1]. According to the product leaflet, leflunomide can determine a number of cutaneous adverse effects, cited as the following: common (increased hair loss, eczema, rash (including maculopapular rash), pruritus, dry skin), less common (urticaria), very rare (TEN, SJS, erythema multiforme), and with unknown frequency (cutaneous lupus erythematosus, pustular psoriasis or worsening psoriasis, DRESS syndrome, skin ulcer) [17].

Reports have documented cases of drug-induced hypersensitivity syndrome [18], leg ulcers [19], phototoxic reactions [5], TEN [6], and cutaneous lupus erythematosus [7].

Leflunomide can cause cutaneous adverse effects through multiple mechanisms: 1. Inhibition of dihydroorotate dehydrogenase (DHODH) and decrease of T-cell proliferation, which may lead to reactivation of conditions like drug-induced lupus or psoriasiform eruptions [11]; 2. Type IV hypersensitivity reactions leading to DIHS/DRESS syndrome, SJS, and TEN [12].

About our cases

Since hydroxychloroquine's most common side effects include mucocutaneous hyperpigmentation, patients may be unaware of them until seeking consultation for another issue. In accordance with statistical data, we encountered a case of hydroxychloroquine-induced mucocutaneous hyperpigmentation (Case 2). Due to prolonged use (about three years) and the inability to discontinue the drug for a therapeutic test, HCQ was deemed the most likely cause. No curative treatment was initiated, as the hyperpigmented lesions were asymptomatic. However, a topical adjuvant hydration treatment was applied, considering that the patient also has Sjögren's syndrome.

In Case 1, annular erythema lesions appeared approximately four months after initiating hydroxychloroquine. No other identifiable cause was found, and treatment with potent topical corticosteroids for two weeks resulted in complete remission. However, the delayed onset of symptoms (four months vs. a few weeks) is inconsistent with typical hydroxychloroquine-induced rashes but remains the most probable cause in our case.

Regarding Case 3, this is the second reported case of phototoxic reaction associated with leflunomide. Sun exposure should be approached with caution, ensuring adequate protection through both physical and topical measures.

Extensive studies on the pathophysiology of hydroxychloroquine- and leflunomide-induced cutaneous adverse effects are lacking. Oxidative stress, phototoxicity, drug accumulation in skin tissues, and immunomodulatory properties may explain these effects, but not all adverse reactions have a well-defined mechanism [7,9]. Future research should focus on elucidating these pathways to improve prevention strategies.

## Conclusions

The use of immunomodulatory and immunosuppressive agents, such as hydroxychloroquine and leflunomide, is essential in the management of connective tissue diseases. However, these medications are associated with a wide range of cutaneous adverse effects, from common reactions like hyperpigmentation, pruritus, and rash to rare but severe conditions such as SJS/TEN, DRESS, and AGEP. The presented cases highlight the importance of recognizing these dermatological manifestations, even when they are not the primary reason for a patient’s visit. Early identification of drug-induced skin reactions can aid in timely intervention, potentially preventing more serious complications. Despite existing knowledge, the exact pathophysiology of these adverse effects remains incompletely understood, warranting further research to better predict, prevent, and manage them. Clinicians should remain vigilant for cutaneous signs in patients undergoing long-term immunomodulatory therapy, ensuring a balance between therapeutic benefits and the risk of adverse effects.
